# TRIM22-Mediated Apoptosis is Associated with Bak Oligomerization in Monocytes

**DOI:** 10.1038/srep39961

**Published:** 2017-01-12

**Authors:** Chi Chen, DongYan Zhao, Shu Fang, QiXing Chen, BaoLi Cheng, XiangMing Fang, Qiang Shu

**Affiliations:** 1 Department of Thoracic and Cardiovascular Surgery, Children’s Hospital, School of Medicine, Zhejiang University, and Zhejiang Key Laboratory for Diagnosis and Therapy of Neonatal Diseases, Hangzhou 310052, China; 2 Clinical Research Center, Children’s Hospital, School of Medicine, Zhejiang University, Hangzhou 310052, China; 3 Department of Anesthesiology, the First Affiliated Hospital, School of Medicine, Zhejiang University, Hangzhou 310003, China

## Abstract

Monocyte apoptosis is a key mechanism that orchestrates host immune responses during sepsis. TRIM22 is constitutively expressed at high levels in monocytes and plays important roles in the antiviral response and inflammation. Overexpression of TRIM22 interferes with the clonogenic growth of monocytic cells, suggesting that TRIM22 may regulate monocyte survival. However, the effect of TRIM22 on monocyte apoptosis remains unknown. In the present report, lipopolysaccharides (LPS)-primed human peripheral blood monocytes expressing higher levels of TRIM22 were more sensitive to apoptosis. This phenomenon was also observed in TRIM22-overexpressing THP-1 monocytes and was associated with the activation of caspase-9 and caspase-3, as well as the increased expression and oligomerization of the pro-apoptotic protein Bak. Similar expression patterns of TRIM22 and Bak were also observed in LPS-primed, apoptotic human peripheral blood monocytes. In addition, the deletion of either the RING domain or the SPRY domain of TRIM22 significantly attenuated TRIM22-mediated monocyte apoptosis and decreased Bak expression and oligomerization. Furthermore, in monocytes from septic patients, TRIM22 levels were down-regulated and positively correlated with Bak levels. Taken together, these results indicate that TRIM22 plays a critical role in monocyte apoptosis by regulating Bak oligomerization and may have a potential function in the pathogenesis of sepsis.

Sepsis triggers a dysregulated host response to infection, which results in life-threatening organ dysfunction^1^,^2^. Monocytes are an important line of host immune defense against microbial infection. They are recruited to the sites of infection, and their prolonged survival triggers immune responses and invading pathogen clearance^3^,^4^. However, prolonged survival of monocytes results in the overproduction of pro-inflammatory cytokines, ultimately leading to tissue and organ damage^3^,^5^. Appropriate monocyte apoptosis terminates their activity and limits inflammation^5^. It is well known that apoptosis of immune effector cells such as lymphocytes plays an important role in sepsis, but data concerning the effects of monocyte survival on sepsis are limited. Previous studies have demonstrated that the outcomes of septic patients are associated with monocyte apoptosis, and this could be improved by regulating monocyte apoptosis^6^,^7^. Thus, revealing a mechanism of monocyte apoptosis may aid in the management of sepsis.

Tripartite motif (TRIM) proteins are an expanding protein family characterized by a conserved tripartite motif, which consists of a RING finger, one or two B-box(es) and an α-helical coiled-coil region^8^. The TRIM proteins participate in diverse biological processes, such as antiviral activities, oncogenesis, cell proliferation and differentiation^9^. Some TRIM family members are also involved in apoptosis^10^. For example, TRIM19 plays an important role in the suppression of cell growth and tumor formation in acute promyelocytic leukemia^11^. In addition, mice and primary cells lacking TRIM19 were rescued from apoptosis induced by various *in vivo* and *in vitro* stimuli^12^,^13^,^14^. Moreover, the expression of a truncated form of TRIM20 *in vivo* led to impaired macrophage apoptosis in a mouse model of familial Mediterranean fever^15^. Other TRIM family members, such as TRIM32 and TRIM35, have also shown pro-apoptotic activity *in vitro*^16^,^17^.

TRIM22 was first identified as an interferon-inducible protein that restricts HIV transcription. TRIM22 is constitutively expressed in peripheral blood leukocytes and lymphoid tissues, such as spleen and thymus^18^. The expression level of TRIM22 in monocytes was 2-fold higher than that in CD4^+^ and CD8^+^ T-lymphocytes and nearly 1.5-fold higher than that in B-lymphocytes^19^. TRIM22 is also a target gene of p53, which is a well-known regulator of cell growth and death. It has been noted that the overexpression of TRIM22 interferes with the clonogenic growth of monocytic U937 cells^20^, suggesting that it may participate in controlling monocyte survival. TRIM22 is also involved in host inflammatory responses^21^. Knockdown of TRIM30, the murine ortholog of TRIM22, increased the expression levels of pro-inflammatory cytokines (TNF-α, IL-6, IL-1β and IL-18). By contrast, the overexpression of TRIM30 protected mice against lipopolysaccharides (LPS)-induced septic shock^22^,^23^. However, whether TRIM30/TRIM22 affects monocyte apoptosis during sepsis remains unknown.

In the present study, the effect of TRIM22 on apoptosis was first investigated in human peripheral blood monocytes and the THP-1 monocytic cell line. We then identified the mechanism by which TRIM22 mediates apoptosis. We also observed how the structure of TRIM22 affects its function. Finally, we measured the expression levels of TRIM22 in septic patients and analyzed the correlations between TRIM22 and apoptosis-associated proteins.

## Results

### Increased endogenous TRIM22 levels in human peripheral blood monocytes were associated with cell apoptosis

**Figure 1:**
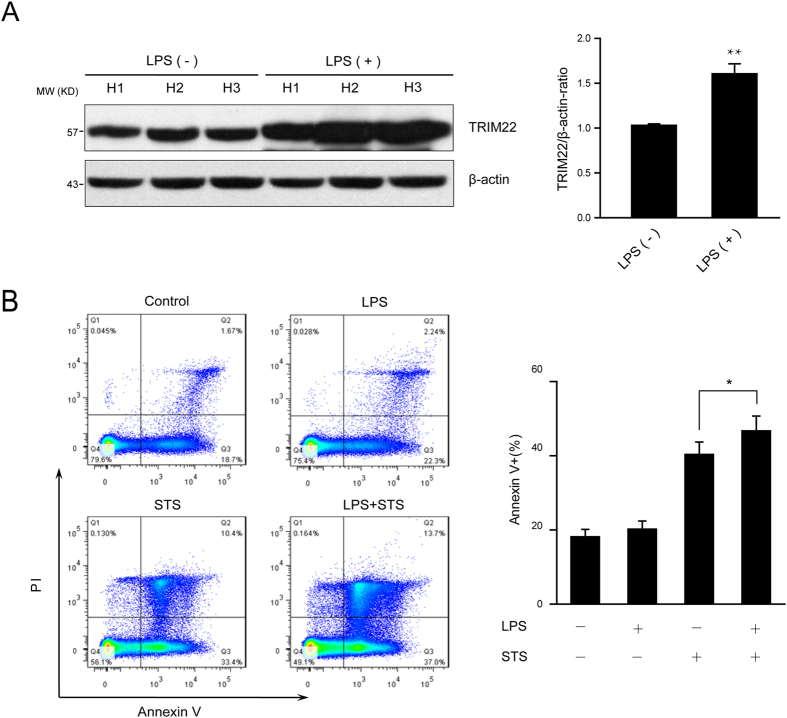
Induced endogenous TRIM22 in human peripheral blood monocytes is associated with cell apoptosis. (**A**) Immunoblot analysis and densitometric quantification of TRIM22 expression in the lysates of human peripheral blood monocytes from three healthy donors in the presence or absence of LPS stimulation (100 ng/ml LPS for 16 h). **P < 0.01. (**B**) LPS-primed human peripheral blood monocytes were challenged with 0.5 μg/ml STS for 8 h before harvest. The cells were stained with Annexin V-fluorescein isothiocyanate and propidium iodide for flow cytometry analysis. Histograms of annexin V^+^ cells are shown, and quantitative data are presented as the mean ± SEM from 3 healthy volunteers. *P < 0.05.

We first investigated whether modulating the expression levels of endogenous TRIM22 could affect monocyte survival. TRIM22 transcription can be induced after LPS stimulation^24^. Here, we found that the protein levels of TRIM22 in human peripheral blood monocytes were upregulated >1.5-fold upon LPS treatment (Fig. 1A). Under these conditions, the upregulation of TRIM22 did not affect monocyte apoptosis, as there were no differences in the proportions of Annexin V^+^ cells between LPS-treated and untreated monocytes. However, when these cells were challenged with staurosporine (STS; an apoptosis inducer), we observed significantly more Annexin V^+^ monocytes in LPS-primed cultures (47.2%) compared with unprimed cultures (40.5%; Fig. 1B). These findings show that monocytes with increased expression levels of TRIM22 are more susceptible to pro-apoptotic stimuli, suggesting a potential role for TRIM22 in mediating monocyte apoptosis.

### Overexpression of TRIM22 sensitized THP-1 cells to apoptosis

**Figure 2:**
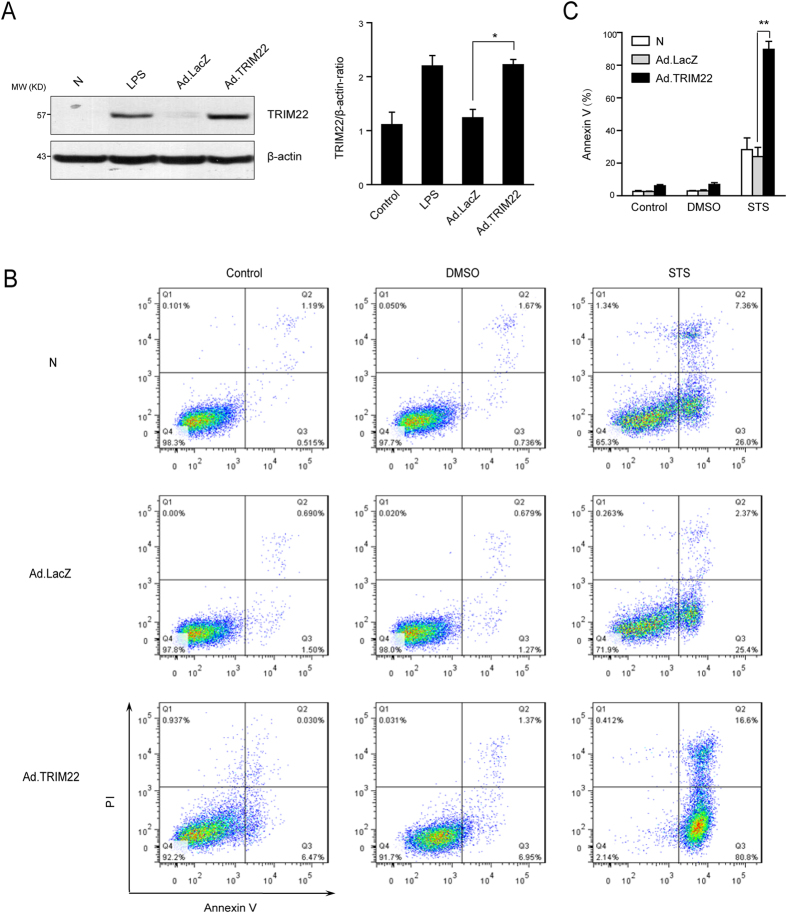
Overexpression of TRIM22 sensitizes THP-1 monocytes to apoptosis. (**A**) The levels of TRIM22 protein in THP-1 cells were analyzed after transduction with recombinant adenoviral vectors for 72 h. *P < 0.05. (**B**) Mock-infected THP-1 cells and THP-1 cells infected with Ad.LacZ or Ad.TRIM22 were treated with 0.5 μg/ml STS or DMSO. Untreated cells were used as controls. After 4 h, cells were stained with Annexin V-fluorescein isothiocyanate and propidium iodide for flow cytometry analysis. (**C**) Histograms of annexin V^+^ cells are shown, and quantitative data are presented as the mean ± SEM from three independent experiments. **P < 0.01.

To confirm our observations, we further investigated the role of TRIM22 in human peripheral blood monocyte apoptosis *in vitro*. To recapitulate the high levels of TRIM22 expression observed in human peripheral blood monocytes after LPS stimulation, a recombinant adenovirus, Ad.TRIM22, was used to overexpress TRIM22 in THP-1 cells. The expression levels of TRIM22 in infected THP-1 cells were about 1.5-fold of those in unstimulated peripheral blood monocytes while comparable to those in LPS-treated peripheral blood monocytes, and were similar to those in LPS-treated THP-1 cells (Fig. 2A). As expected, when the cells were subjected to pro-apoptotic stimuli, TRIM22-overexpressing cells displayed increased levels of apoptosis compared with the control (Ad.LacZ-transduced) and mock-transduced cells (Fig. 2B and C).

### TRIM22 mediated apoptosis through a caspase-9-dependent pathway

**Figure 3:**
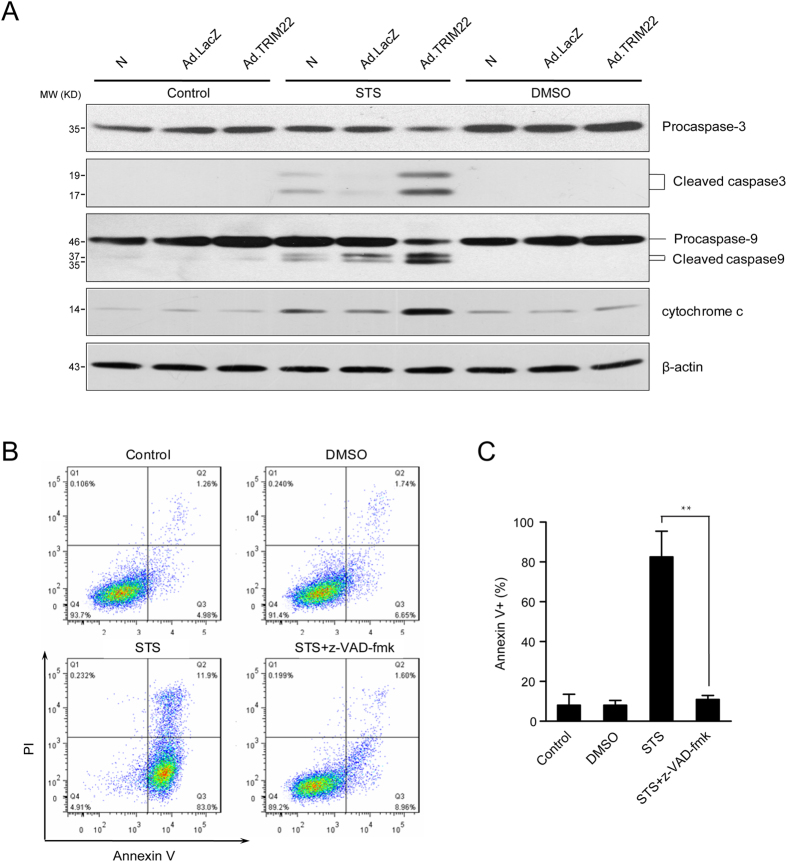
TRIM22 sensitizes monocytes to apoptosis in a caspase-dependent manner. (**A**) Cells were exposed to 0.5 μg/ml STS or DMSO, or left untreated for 4 h, and were then analyzed by Western blotting. TRIM22 increased the cleavage of caspase-3 and caspase-9 and enhanced the release of cytochrome *c*. (**B**) TRIM22-overexpressing THP-1 cells were pre-incubated with the pan-caspase inhibitor Z-VAD-FMK (100 μM) for 1 h and then challenged with 0.5 μg/ml STS for 4 h. Apoptosis was analyzed by flow cytometry. (**C**) Histograms of annexin V^+^ cells are shown, and quantitative data are presented as the mean ± SEM from three independent experiments. **P < 0.01.

Next, we determined whether TRIM22-induced apoptosis was dependent upon caspase activity. In TRIM22-overexpressing cells, procaspase-3 and procaspase-9 were cleaved, and activated forms of caspase-3 and caspase-9 were elevated after STS challenge (Fig. 3A). STS-induced cytochrome *c* release was also increased in TRIM22-overexpressing cells (Fig. 3A). Moreover, when TRIM22-overexpressing cells were treated with the pan-caspase inhibitor Z-VAD-FMK, STS-induced apoptosis was completely abolished (Fig. 3B and C). These findings demonstrate that TRIM22 sensitizes monocyte to apoptosis in a caspase-dependent manner.

### Overexpression of TRIM22 modulated Bak expression and oligomerization

**Figure 4:**
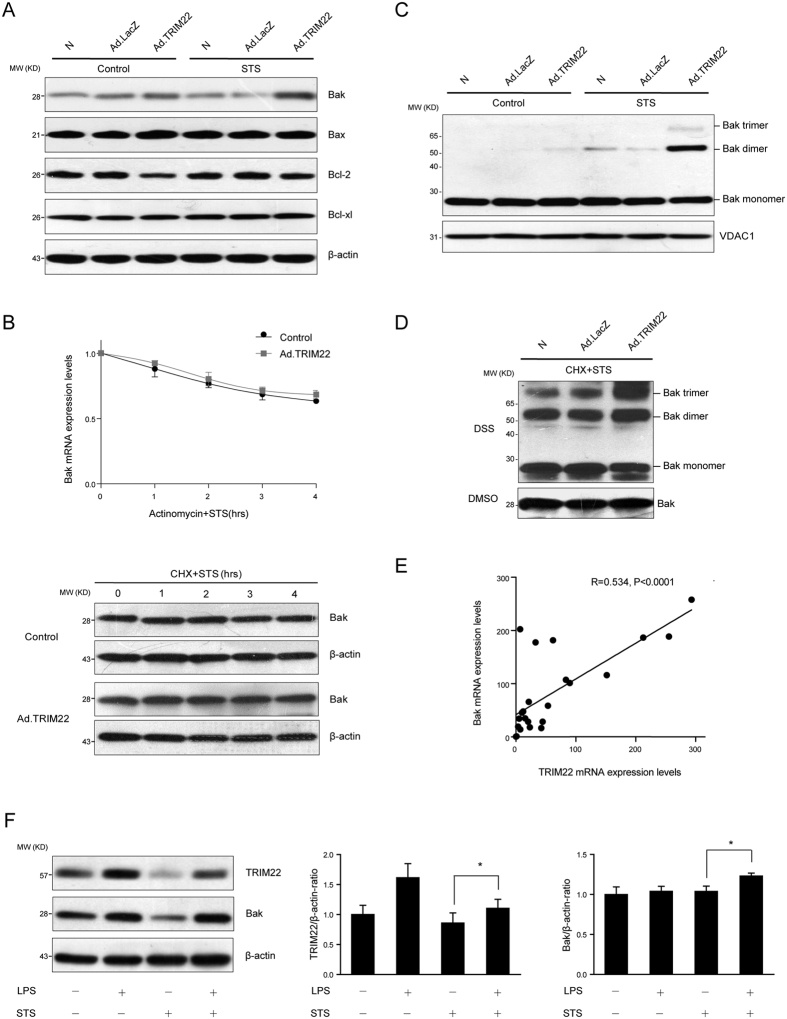
TRIM22 upregulation increases Bak expression and oligomerization. (**A**) TRIM22-induced alterations in protein levels of Bcl-2 family members are illustrated using Western blots. (**B**) TRIM22-overexpressing THP-1 cells were pre-treated with 10 μg/ml actinomycin or 20 μg/ml CHX for 1 h and then challenged with STS for 4 h. The cells were harvested after STS challenge at the indicated time points. The mRNA and protein levels of Bak were then assessed. (**C**) THP-1 cells were treated with 0.5 μg/ml STS for 4 h and then incubated with 4 mM disuccinimidyl suberate (DSS). The multimer conformation of Bak was visualized by immunoblotting. VDAC1 was used as a mitochondrial loading control. (**D**) Cells were pre-treated with CHX (20 μg/ml) for 1 h to block Bak protein synthesis. Oligomerization of Bak was examined as indicated. Non cross-linked Bak incubated with DMSO control buffer was run as a monomer. (**E**) Monocytes isolated from 23 healthy volunteers were exposed to 100 ng/ml LPS for 16 h, treated with 0.5 μg/ml STS for 8 h before harvest. Correlations between the expression levels of TRIM22 and Bak mRNA were analyzed (r = 0.534, *P* < 0.0001). (**F**) Representative immunoblots and densitometric quantifications illustrating protein levels of TRIM22 and Bak in monocytes. TRIM22 and Bak levels were normalized to β-actin. Data are presented as the mean ± SEM from three healthy volunteers. *P < 0.05.

We next investigated whether TRIM22-mediated apoptosis induced changes in other proteins involved in the intrinsic apoptosis pathway. The basal expression levels of Bcl-2 in TRIM22-overexpressing THP-1 cells were lower than those in mock- and Ad.LacZ-transduced cells, but were not further suppressed upon STS stimulation. Interestingly, we found that the expression levels of Bak were significantly upregulated following STS treatment in TRIM22-overexpressing cells (Fig. 4A). This upregulation of Bak was not associated with changes in the half-life of Bak because the inhibition of transcription or translation did not affect levels of Bak expression (Fig. 4B).

Bak forms large oligomeric complexes that trigger cytochrome *c* release from the mitochondria^25^,^26^. After the induction of apoptosis, we observed more Bak oligomers in TRIM22-overexpressing THP-1 cells (Fig. 4C). To examine whether TRIM22 promoted Bak oligomerization directly or was dependent on increased Bak protein synthesis during apoptosis, we evaluated the oligomerization in apoptotic cells pretreated with the protein synthesis inhibitor cycloheximide. After treatment, increased Bak oligomerization in TRIM22-overexpressing cells was still observed (Fig. 4D), demonstrating that TRIM22 induces Bak oligomerization.

The relationship between TRIM22 and Bak was further studied in LPS-primed, STS-challenged peripheral blood monocytes from healthy volunteers. Correlation analysis showed a positive correlation between the expression levels of TRIM22 and Bak (*r* = 0.534, *P* < 0.0001) (Fig. 4E). Moreover, upon STS challenge, higher Bak protein levels were observed in LPS-primed peripheral blood monocytes, which have already been shown to demonstrate inducible TRIM22 expression (Fig. 4F). These findings further demonstrate a critical role for the pro-apoptotic protein Bak in TRIM22-mediated apoptosis.

### RING and SPRY domains were involved in TRIM22-mediated monocyte apoptosis

**Figure 5:**
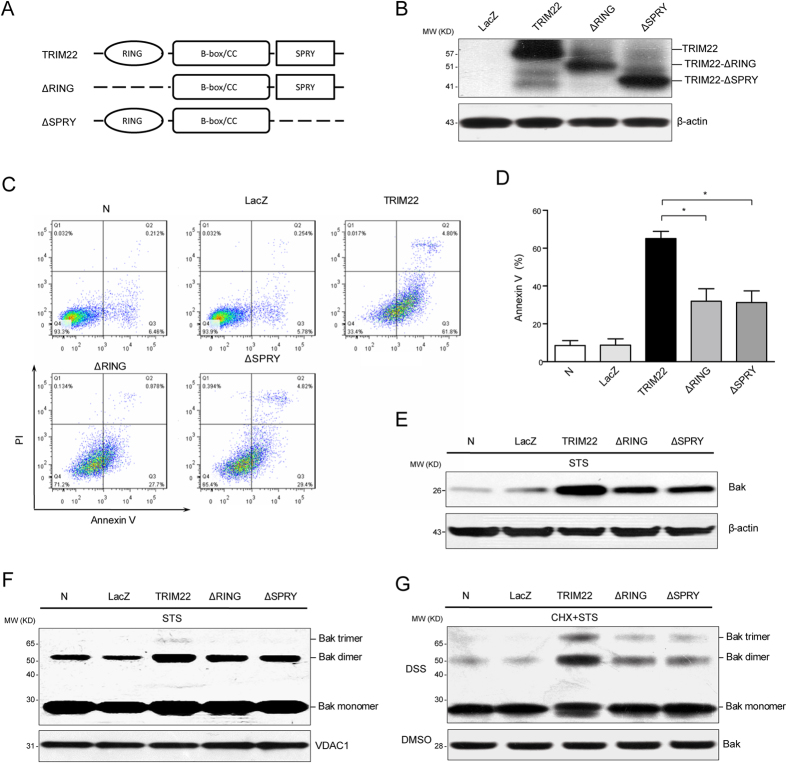
The RING and SPRY domains of TRIM22 are associated with the sensitization of TRIM22-overexpressing monocytes to apoptosis. (**A**) Schematic of wild-type TRIM22 and domain-deletion mutants. (**B**) THP-1 cells were transduced with adenoviruses carrying wild-type TRIM22 or domain-deletion mutants. After 72 h, the expression levels of the relevant proteins were measured using Western blots. (**C**) Mock-infected cells or cells infected with wild-type TRIM22, domain-deletion mutants or control adenoviruses were treated with 0.5 μg/ml STS for 4 h, and apoptosis was analyzed using flow cytometry. (**D**) Histograms of annexin V^+^ cells are shown, and quantitative data are presented as the mean ± SEM from three independent experiments. *P < 0.05. The expression levels (**E**) and oligomerization of Bak (**F**) were measured using Western blots. VDAC1 was used as a mitochondrial loading control. (**G**) THP-1 cells were pre-treated with 20 μg/ml CHX for 1 h and oligomerization of Bak was analyzed as described above.

Previous studies have shown that the RING and SPRY domains play key roles in TRIM22 function^27^,^28^. To determine whether these domains were involved in the pro-apoptotic role of TRIM22, we constructed recombinant adenoviruses expressing domain-deletion mutants, including TRIM22-ΔRING and TRIM22-ΔSPRY (Fig. 5A and B), and we evaluated their effects on STS-induced apoptosis. Deletion of either the RING or SPRY domain moderately increased cell apoptosis (Fig. 5C and D), and partially blocked Bak expression (Fig. 5E) and oligomerization (Fig. 5F). Furthermore, oligomerization of Bak was not influenced by cycloheximide treatment (Fig. 5G).

### TRIM22 expression levels positively correlated with Bak expression in monocytes from septic patients

**Figure 6:**
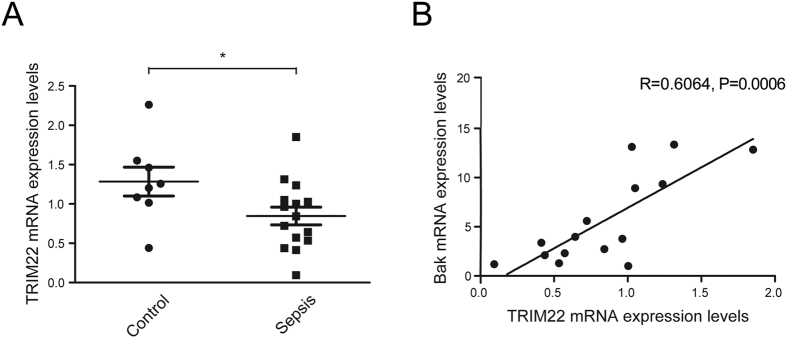
TRIM22 levels in monocytes from septic patients are positively correlated with Bak levels. The expression levels of TRIM22 and Bak were analyzed using quantitative real-time polymerase chain reaction with the housekeeping gene β-actin as an internal control. (**A**) TRIM22 mRNA levels in monocytes isolated from septic patients and controls. Dots represent individual subjects and data are presented as the mean ± SEM. *P < 0.05. (**B**) Correlation of TRIM22 and Bak mRNA levels in monocytes from septic patients (r = 0.6064, *P* = 0.0006).

We further measured the expression levels of TRIM22 and Bak in peripheral blood monocytes collected from septic patients. The demographic and clinical characteristics of enrolled septic patients are listed in Table 1. The septic group consisted of 15 patients, including five patients with pneumonia, four patients with hepatobiliary infection, two patients with peritonitis, two patients with pancreatitis, and two patients with soft-tissue infection. Infection was documented in all patients by microbiologic inspection. The control subjects consisted of 8 non-septic, critically ill surgical patients (age 54.1 ± 17.7 years; 4 females, 4 males). Real-time quantitative PCR analysis showed that TRIM22 mRNA levels in the septic patients were significantly lower compared with those in the non-septic controls (Fig. 6A). Furthermore, TRIM22 mRNA levels were positively correlated with Bak levels in septic patients (*r* = 0.6064, *P* = 0.0006; Fig. 6B). Since Bak is an important pro-apoptotic protein, these findings indicate that in sepsis, monocyte survival may be autoregulated via the control of Bak-associated TRIM22 expression.

## Discussion

In this study, increasing the levels of both endogenous and exogenous TRIM22 sensitized monocytes to STS-induced apoptosis. This function of TRIM22 was related to the increased expression and oligomerization of Bak via a caspase-dependent pathway and was associated with the RING and SPRY domains of the TRIM22 molecule. In addition, the mRNA levels of TRIM22 were down-regulated and positively correlated with Bak transcripts in monocytes from septic patients.

As a p53 target gene, TRIM22 inhibits the clonogenic growth of U937 monocytic cells^20^. In this study, we found that LPS-primed monocytes expressing high levels of TRIM22 were more sensitive to STS-induced apoptosis. In addition, the recombinant adenovirus-mediated overexpression of TRIM22 enhanced the susceptibility of monocytes to STS-induced apoptosis. In light of previous studies regarding the role of TRIM22 in antiviral immunity, cytokine production, and inflammatory diseases, our findings suggest that TRIM22 may mediate inflammation by controlling monocyte survival.

Previous studies have shown that TRIM19 is required for the activation of caspase-1 and caspase-3 in mouse splenocytes, suggesting that TRIM19 is involved in caspase-dependent apoptosis^13^. By contrast, the overexpression of TRIM19 induced apoptosis in rat embryonic fibroblasts in the absence of caspase-3 activation, and the caspase inhibitor Z-VAD-FMK failed to block TRIM19-induced cell death^14^. These data reveal that TRIM proteins can participate in apoptosis via caspase-dependent or -independent pathways, potentially in a cell type-specific manner. In our study, TRIM22 overexpression promoted the cleavage of procaspase-9 and procaspase-3, elevated the expression levels of cleaved caspase-3 and cleaved caspase-9, and enhanced cytochrome *c* release in STS-challenged monocytic cells. Moreover, pretreatment with the caspase inhibitor Z-VAD-FMK effectively inhibited TRIM22-mediated apoptosis. Together, these findings demonstrate that TRIM22 promotes monocyte apoptosis via a caspase-dependent pathway.

p53 is an important regulator of cell growth suppression and apoptosis. p53 induces apoptosis by regulating the transcription of pro-apoptotic and anti-apoptotic genes such as Bax and Bcl-2^29^. Given that TRIM22 is a target gene of p53, we propose that TRIM22 promotes monocyte apoptosis by regulating the Bcl-2 family proteins. Following STS challenge, the overexpression of TRIM22 significantly enhanced Bak expression but did not affect the expression of other Bcl-2 family proteins such as Bax and Bcl-xl. This initial finding was further confirmed in STS-challenged LPS-primed human peripheral blood monocytes, in which both mRNA and protein levels of Bak were positively correlated with those of TRIM22. These data suggest a critical role for Bak in TRIM22-sensitized apoptosis.

In stressed cells, inactive Bak undergoes an activating conformational change leading to the formation of higher-order multimers, followed by oligomerization. Bak oligomerization enhances the permeabilization of the outer mitochondrial membrane, which results in the release of pro-apoptogenic factors (such as cytochrome *c*) from the mitochondria into the cytosol^26^,^30^. In addition, mitochondrial p53 can interact with Bak, leading to Bak oligomerization and cytochrome *c* release^31^,^32^. In the current study, we observed more oligomerization of the Bak protein in TRIM22-overexpressing monocytes independent of protein synthesis, suggesting a role for TRIM22 in apoptosis-associated Bak oligomerization. This may also explain the sensitization to apoptosis in LPS-treated monocytes expressing higher levels of TRIM22. However, whether TRIM22-mediated apoptosis was caused by Bak oligomerization or triggered via other pathways remains unknown and requires further investigation.

TRIM22 contains a conserved RBCC structure beginning with a RING domain at the N-terminus and followed by a B30.2/SPRY domain at the C-terminus^33^. Previous studies have demonstrated that these distinct domains mediate the diverse functions of TRIM22. The RING domain is important for the E3 ubiquitin ligase activity of TRIM proteins^34^, which is associated with the effects of TRIM family members on cell survival via the ubiquitination and proteasomal degradation of p53 or other apoptosis-related proteins^12^,^16^,^35^,^36^,^37^. The function of the SPRY domain is not well understood, although several studies suggest it may mediate protein-protein interactions^38^,^39^. Both the RING and SPRY domains of TRIM22 are essential for TRIM22-mediated anti-HBV activity and the activation of NF-κB^40^,^41^. In this study, using domain-deletion mutants, we found that deletion of either the RING domain or the SPRY domain significantly attenuated STS-induced apoptosis, which was associated with decreased Bak expression and oligomerization. However, what would both domains being so different only partially and equally contribute to change Bak expression and oligomerization or whether the observations are attributed the truncated peptides but not the specific domain truncated remians unclear. Further additional experiments to dissect these mechanisms should help us understand well.

Sepsis induces a multitude of defects in immunity that causes aberrant inflammation, immune suppression, susceptibility to infections, and death. One of the manifestations of sepsis-induced immunosuppression is monocyte/macrophage dysfunction^42^. Monocytes/macrophages are important players in the pathogenesis of sepsis. Monocytes/macrophages from septic patients undergo functional reprogramming from a proinflammatory to an immunosuppressive phenotype^43^. The proinflammatory response often predominates in the early phase of an infection. And most patients will rapidly progress to an immunosuppressive state, characterized by decreased phagocytic ability, reduced bactericidal activity, and attenuated proinflammatory cytokine production^42^. As TRIM22 expression was induced upon LPS challenge in human peripheral blood monocytes from healthy donors, decreased TRIM22 levels in septic patients might result from immunosuppression.

Previous studies have demonstrated that the overexpression of TRIM30, the mouse ortholog of TRIM22, protected mice from LPS-induced septic shock^22^,^23^. Although these studies did not focus on the effects of TRIM30 on monocyte apoptosis, given the present findings, it is reasonable to speculate that TRIM30 might also improve outcomes in septic mice through the sensitization of monocytes to apoptosis.

In conclusion, we show that TRIM22 sensitizes monocytes to STS-induced apoptosis. Upregulation of TRIM22 triggers the expression and oligomerization of Bak and subsequently leads to cytochrome *c* release in a caspase-9- and caspase-3-dependent manner. Both the RING domain and the SPRY domain of the TRIM22 molecule are associated with its pro-apoptotic function. These findings not only illustrate the role of TRIM22 in monocyte apoptosis but also indicate the potential functions of TRIM22 in inflammatory diseases such as sepsis.

## Methods

### Study subjects and data collection

Patients admitted to the Intensive Care Unit at the First Hospital of Zhejiang University (Hangzhou, Zhejiang, China) from October 2014 to February 2015 were enrolled in the study. All septic patients fulfilled the recommended criteria of the American College of Chest Physicians and Society of Critical Care Medicine Consensus Conference^44^. Patients younger than 18 years of age, those with an immunological disease, organ transplantation, terminal illness, or those receiving corticosteroids or chemotherapy were excluded. The following data were collected from each septic patient: age, sex, the length of ICU stay, mortality, Acute Physiology and Chronic Health Evaluation II (APACHE II) score at admission, and Sequential Organ Failure Assessment (SOFA) score. In addition, 8 control subjects and 26 healthy blood donors were included in the study. The study protocol was performed in accordance with the Declaration of Helsinki. The Institutional Review Board (the Ethics Committee of the First Hospital of Zhejiang University, Hangzhou, Zhejiang, China) reviewed and approved all procedures (reference no. 2014319). The methods were carried out in accordance with the approved guidelines. Written informed consent was obtained from the patients or their relatives.

### Blood sampling

Blood samples were collected into tubes containing ethylenediaminetetraacetic acid within 24 h after diagnosis of sepsis. Peripheral blood mononuclear cells were separated using Ficoll-Hypaque density gradient centrifugation at 2000 rpm for 20 min at room temperature. For monocyte isolation, peripheral blood mononuclear cells were allowed to adhere for 2 h at 37 °C in RPMI1640 medium containing 10% fetal bovine serum. After suspension cells were removed, adherent monocytes were collected for the following experiments.

### Real-time quantitative PCR

Total RNA was isolated using TRIzol reagent (Invitrogen, Carlsbad, California, USA). Reverse transcription was performed with 1 μg of RNA using a Reverse Transcription System kit (Promega, Madison, Wisconsin, USA), according to the manufacturer’s instructions. Quantitative PCR was carried out with an ABI Prism 7500 system (Applied Biosystems, Carlsbad, California, USA) using the SYBR Premix Ex Taq^TM^ kit (Takara, Shiga, Otsu, Japan). PCR was performed with the following primers: TRIM22: Forward: 5′-AGAAGCTGGAAGATGACATCA-3′, Reverse: 5′-AGCTGCTGCCAGGTTATC-3′; Bak: Forward: 5′-ACCCAGAGATGGTCACCT TA-3′, Reverse: 5′-GTCGGTTGATGTCGTCC-3′; and β-actin: Forward: 5′-GATGGGCACAGTGTGGGTGACCC-3′, Reverse: 5′-TGGAGAAAATCTGGCACCACACC- 3′. The expression levels of target genes were normalized to the housekeeping gene β-actin.

### Cell culture

THP-1 and HEK293 (human embryonic kidney) cells were purchased from the American Type Culture Collection (Manassas, Virginia, USA). AD293 cells (adenoviral E1-transformed human embryonic kidney cell) were a kind gift from Prof. Hangping Yao (the First Hospital of Zhejiang University, Hangzhou, Zhejiang, China). THP-1 cells were propagated in RPMI-1640 medium supplemented with 10% fetal bovine serum, 100 U/ml penicillin, and 100 μg/ml streptomycin. AD293 cells and HEK293 cells were cultured in Dulbecco’s modified Eagle’s high-glucose medium supplemented with 10% fetal bovine serum, 100 U/ml penicillin and 100 μg/ml streptomycin. The cells were maintained at 37 °C in a 5% CO_2_ atmosphere.

### Recombinant adenoviral vectors

The recombinant replication-deficient adenoviral vector Ad.TRIM22, its RING domain-deletion mutant (Ad.TRIM22-ΔRING), its SPRY domain-deletion mutant (Ad.TRIM22-ΔSPRY), and a control vector (Ad.LacZ) were constructed as previously described^45^. The adenoviruses were amplified in AD293 cells, and the viral titers (pfu/ml) were determined using a plaque-forming unit assay with HEK293 cells.

### Adenoviral transduction

THP-1 cells (1 × 10^6^/ml) were transduced with recombinant adenovirus at multiplicities of infection of 200 in serum-free medium. After incubation at 37 °C for 2 h, fetal bovine serum was added to a final concentration of 10%. After an additional culture period of 72 h, cells were harvested, and the expression levels of TRIM22 and deletion mutants were analyzed.

### Cell treatment

Monocytes isolated from healthy volunteers were exposed to 100 ng/ml LPS (*Escherichia coli* 0111:B4; Sigma, St. Louis, Missouri, USA) for 16 h and treated with 0.5 μg/ml STS (Enzo Life sciences, Farmingdale, New York, USA) for 8 h prior to harvest. Apoptosis was induced in transduced and control THP-1 cells with 0.5 μg/ml STS for 4 h in complete medium. To inhibit caspase activity, cells were pretreated with 100 μM Z-VAD-FMK (Sigma, St. Louis, Missouri, USA) for 1 h prior to STS treatment. To inhibit the mRNA and protein synthesis of Bak, cells were treated with 10 μg/ml actinomycin or 20 μg/ml CHX (Sigma, St. Louis, Missouri, USA), respectively, 1 h before STS stimulation.

### Flow cytometry analysis

After treatment, THP-1 cells were harvested and washed twice with phosphate-buffered saline. The apoptotic cells were labeled using an Annexin V-fluorescein isothiocyanate apoptosis detection kit (Biouniquer, Hong Kong, China) according to the manufacturer’s instruction. Briefly, the cell pellet was resuspended in 500 μl of Annexin V-fluorescein isothiocyanate binding buffer. Five microliters of annexin V-fluorescein isothiocyanate and propidium iodide were then added, and the cells were incubated for 10 min at room temperature. Samples were analyzed on an LSR II flow cytometer (BD Biosciences, Franklin Lakes, New Jersey, USA). Data analysis was performed with FlowJo software.

### Western blotting

Harvested cells were lysed in ice-cold radioimmunoprecipitation buffer (Beyotime, Shanghai, China) containing 1 mM phenylmethylsulfonyl fluoride for 40 min at 4 °C. The lysates were collected by centrifugation at 14,000 rpm for 15 min at 4 °C. Cytoplasmic proteins were extracted as previously described^46^. Protein concentration was quantified using a BCA protein assay kit (Pierce, Rockford, Illinois, USA). Proteins (20 μg) were separated on a 12% NuPAGE Bis-Tris gel (Novex, San Diego, California, USA) and blotted onto polyvinylidene difluoride membranes (Millipore, Billerica, Massachusetts, USA). The membranes were blocked with 5% skim milk in Tris-buffered saline with 0.05% Tween-20 for 1 h at room temperature, incubated overnight with specific primary antibodies, washed three times with Tris-buffered saline containing 0.05% Tween-20, and further incubated for 1 h with appropriate horseradish peroxidase-conjugated secondary antibodies (Jackson ImmunoResearch, West Grove, Pennsylvania, USA). After washing the membranes with Tris-buffered saline containing 0.05% Tween-20, the protein bands were visualized with an EZ-ECL kit (Bioind, Kibbutz, Beit Haemek, Israel). The rabbit-derived primary antibodies included Bcl-2, Bcl-xL, Bak, Bax, and cytochrome *c* (all from Epitomics, Inc., Burlingame, California, USA), as well as cleaved caspase-3 and caspase 9 (Cell Signaling Technology, Inc., Beverly, Massachusetts, USA). A mouse anti-β-actin monoclonal antibody (Sigma, St. Louis, Missouri, USA) was used as a loading control.

### Cross-linking

The Bak oligomerization assay was performed as previously reported^47^. Briefly, mitochondria were isolated and incubated with 4 mM disuccinimidyl suberate (Sigma, St. Louis, Missouri, USA) for 30 min at room temperature. Cross-linked samples were analyzed by western blotting using an anti-Bak antibody (Epitomics, Burlingame, California, USA). Rabbit anti-VDAC1 monoclonal antibodies (Abcam, Cambridge, Massachusetts, USA) were used as a mitochondrial loading control.

### Statistical analysis

Data are presented as the mean ± SEM. Statistical significance among groups was assessed by One-way ANOVA using GraphPad Prism 5.0 (GraphPad Software Inc., La Jolla, California, USA). Bonferroni’s test was used to correct for multiple comparisons where applicable. The relationship between the expression levels of TRIM22 and Bak was assessed using the Spearman correlation test. Differences were considered statistically significant when a two-tailed *P* value was less than 0.05.

## Additional Information

**How to cite this article**: Chen, C. *et al*. TRIM22-Mediated Apoptosis is Associated with Bak Oligomerization in Monocytes. *Sci. Rep.* **7**, 39961; doi: 10.1038/srep39961 (2017).

**Publisher's note:** Springer Nature remains neutral with regard to jurisdictional claims in published maps and institutional affiliations.

## Figures and Tables

**Table 1:** Patient Characteristics.

Characteristics	Sepsis (n = 15)
Age (yrs)	58.9 ± 16.7
Sex, male (%)	9 (60%)
APACHE II score	23.9 ± 9.1
SOFA score	10.1 ± 5.2
Sepsis due to:	
pneumonia	5 (33.3%)
hepatobiliary systemic infection	4 (26.7%)
peritonitis	2 (12.3%)
pancreatitis	2 (12.3%)
soft-tissue infection	2 (12.3%)
Length of ICU stay	12.9 ± 9.8
Length of hospital stay	17.7 ± 11.8
ICU mortality rate (%)	3 (20%)
